# Body mass index and peer victimization: A transactional model

**DOI:** 10.1002/ab.21942

**Published:** 2020-12-17

**Authors:** Niharika Thakkar, Mitch van Geel, Maike Malda, Ralph C. A. Rippe, Paul Vedder

**Affiliations:** ^1^ Department of Education and Child Studies Leiden University Leiden The Netherlands; ^2^ Amsterdam UMC Vrije Universiteit Amsterdam Amsterdam The Netherlands; ^3^ Department of Public Health and Primary Care Cambridge University Cambridge UK

**Keywords:** adolescent, body mass index, India, transactional model, victimization

## Abstract

Past research has shown concurrent associations between adolescent's body mass index (BMI) and classroom bullying victimization experiences. The goal of this three‐wave longitudinal study is to examine a transactional model of associations between BMI and bullying victimization among adolescents in India. We investigate concurrent unidirectional and bidirectional relations between BMI and victimization. In a sample of 1238 students from nine schools (Grades 7–9; *M*‐age_T1_ = 13.15, *SD* = 1.16) in Indore, India, we used self‐ and peer‐reports to measure bullying victimization in the classroom, and objective measurement of students' height and weight to collect data on adolescents' BMI, across three waves in one school year. Structural equational modeling was used to test transactional relations between BMI and bullying victimization. For self‐reported victimization, there was no concurrent or over time association between BMI and victimization for boys or girls in the present study. For peer‐reported victimization, we observed concurrent associations between BMI and victimization for boys and girls and a prospective relation where higher BMI corresponded to less victimization over time for boys. The study yielded mainly concurrent relations between BMI and victimization among adolescents in India. Results from western countries may not generalize to India.

## 1 INTRODUCTION

Worldwide there has been an increase in the prevalence of obesity among school‐going children (Li et al., 2020; WHO, 2018). Body mass index (BMI) is the weight‐to‐height ratio, where an increased BMI, specific to growth standards of a population, is indicative of overweight and obesity in the population (WHO, 2018). Being obese or overweight among youth are associated with a plethora of adverse physical, psychosocial, and psychological consequences (Li et al., 2020; Sheinbein et al., 2019), one of them being increased risk of being bullied (Van Geel et al., 2014; Waasdorp et al., 2018). However, these results were mostly based on cross‐sectional designs that do not allow for analysis of directionality of relations, and the few longitudinal studies that have examined the direction of links have reported inconsistent results (Adams & Bukowski, 2008; Baldwin et al., 2016; Lee et al., 2018; Lumeng et al., 2010). The purpose of the present study is to examine directions in the relation between BMI and victimization, by using a transactional model with data from a three‐wave longitudinal study in an urban area in India.

Bullying is a repetitive and intentional act of aggression, in which the aggressor is more powerful than the victim (Olweus, 1993). Investigating the direction of the relation between victimization and BMI raises the question of whether adolescents who are obese or overweight are more likely to be victimized, or whether bullied youth run a risk of becoming obese or overweight. On the one hand, past literature reports that weight status plays an active role as a predictor of bullying within the school environment (Janssen et al., 2004; Pearce et al., 2002; Waasdorp et al., 2018). In a longitudinal study (Lumeng et al., 2010), 8–11‐year‐old children who were obese in the US were found to be more likely to be bullied as compared to their nonobese counterparts. In many contemporary societies, there is a prevailing stigma attached to children who are obese and overweight that they are lazy, clumsy, or lacking in willpower (Brewis et al., 2018), and, thus, deviating from appearance ideals could put them at a higher risk of being bullied (Brixval et al., 2012).

Contrastingly, weight‐based bullying may trigger a disturbed emotional state, which, in turn, may lead to unhealthy eating habits like binge eating or emotional eating for comfort (Brewis et al., 2018; Copeland et al., 2015). Bullying victimization reinforces an adolescent's negative feelings of self‐concept concerning the appearance, which leads to depression that in turn results in an increased BMI (Adams & Bukowski, 2008; Lee & Vaillancourt, 2018). Furthermore, depressed mood or decreased self‐esteem, which are both commonly found to be associated with bullying victimization (Van Geel et al., 2018), may be precursors of obesity on account of a shared neuroendocrine pathway between depression and obesity.

In the present study, we propose that the relationship between bullying victimization and weight status could be studied using a reciprocal‐effect or transactional model (Sameroff, 2009). This model indicates a circular path of influence between BMI and victimization, where instead of a unidirectional influence running from BMI to victimization or victimization to BMI, the two factors mutually co‐construct each other in a bidirectional capacity (Adams & Bukowski, 2008; Mamun et al., 2013; Midei & Matthews, 2011). One particular longitudinal study in the recent past that examined transactional associations between peer victimization and BMI found that peer victimization had direct and indirect effects on BMI across a 2–4‐year period proving that peer victimization poses a significant risk to increased BMI, but also suggesting reciprocal links between victimization and BMI via body dissatisfaction (Lee & Vaillancourt, 2019). In the present study, BMI and victimization are the two constructs that make the context of the model, whereas the direction of influence between the two constructs, whether unidirectional, bidirectional, or reciprocal, refers to the structure of the model. The model builds on previous research by going beyond the examination of the influence of BMI on victimization, to also describe how victimization, in turn, affects BMI, representing a dynamic and cyclical process in which the two constructs are in constant flux, and continually redefined by the ongoing process, in a group of non‐western Indian youth.

Even though India is one of the fastest‐growing economies in the world at present, poverty, food insecurity, and malnourishment persist (Jaacks et al., 2015), but also western lifestyles and food habits are increasingly influencing Indian society (Brewis et al., 2018; Kalra et al., 2012; Misra et al., 2011; WHO, 2018). As a consequence, there is a double burden of malnourishment as well as increasing obesity in India (Yang et al., 2019). Inadequate nutrition during infancy and childhood, followed by exposure to high‐fat, energy‐dense, micronutrient‐poor foods combined with a lack of physical activity during adolescence, has made it common to find undernutrition and obesity existing side by side within the same community, sometimes even the same households in India (WHO, 2018). Furthermore, there is a scarcity of research on the topic of victimization from India, a country with 236 million youth, the largest number by country worldwide (UNFPA, 2014). Moreover, research with longitudinal designs to examine bidirectional associations between BMI and victimization through a reciprocal effect model is completely lacking in India. The present study focuses on cultural replication and cross‐validation of research in the field of BMI and victimization to determine the extent of generalizability of previous global findings in India (Thakkar et al., 2020).

A significant limitation observed in most past studies is the use of self‐reports to measure students' victimization experiences (Adams & Bukowski, 2008; Fox & Farrow, 2009; Mamun et al., 2013), and also to measure height and weight (Lee & Vaillancourt, 2019). Self‐reports run the risk of being biased by individual predispositions and shared method variance (Lee et al., 2018). Peer‐reports more accurately provide a perspective from a larger group of direct observers (Malamut et al., 2020). In the present study, the construct of victimization is measured using peer‐reports as well as self‐reports, assuring better validity of the construct measured (Van Geel et al., 2017). We also use objective measures of height and weight because self‐reported data on BMI may be influenced by the student's desirability to adhere to social norms of weight resulting in under‐reporting of weight out of shame, or due to students' lack of information about their own up‐to‐date measurements (Chau et al., 2013).

Furthermore, we assess students' victimization experiences in the classroom, and their weight (BMI) at three time points during 1 school year. This design allows an analysis of a transactional model. Building on previous literature (Adams & Bukowski, 2008; Mamun et al., 2013), we hypothesize that there will be concurrent two‐term unidirectional relations (i.e., BMI predicts victimization, or victimization predicts BMI) as well as three‐term reciprocal relations (i.e., BMI predicts victimization experiences, and these experiences, in turn, are related to an increase in BMI; also victimization predicts BMI and this leads to further victimization of adolescents). In addition, given the differences observed in victimization experiences between boys and girls (Griffiths et al., 2006; Thakkar et al., 2020), the present study investigates differences in the association between BMI and victimization by conducting separate analyses for boys and girls. Multigroup model testing pathways across genders to compare differences between boys and girls within the same model, although interesting, was deemed as beyond the scope of the present research in the interest of the brevity of the study and reporting of results.

## 2 METHODS

The study reported here is part of a larger project on bullying and victimization in Indian schools. This dataset has previously been used in a publication about psychopathy and bullying (Thakkar et al., 2019). Here, we present only the variables relevant to the current paper.

### 2.1 Participants

Data were collected from nine schools in and around the city of Indore in central India at three time points with intervals of 3 months in the school year of 2015–2016. A total of 1238 students (Grades 7–9) were included in the analyses (1120 at T1—296 girls, 824 boys; 1036 at T2—274 girls, 762 boys; and 1006 at T3—282 girls, 724 boys). Students completed the questionnaire in either Hindi (*N* = 497; 40%), or English (*N* = 741; 60%), depending on the formal language of instruction of the participating schools. Of the nine participating schools, three were public schools (i.e., funded and run by the government) whereas six were private schools (privately owned by nongovernmental organizations). Eight schools were mixed boys' and girls' schools, whereas one school was an all‐boys school.

Large class sizes with sometimes over 50 students sitting closely together, combined with laxed disciplinary structures have long been identified to complicate data collection processes in India (Bapat, 2016). The current study is also affected by this, and, therefore, some exclusions in data were made to eventually maintain a sample that is consistent with global research standards. The initial sample consisted of 1908 students from 10 schools, between the ages of 11–16 years, from Grades 7–9 (*M* age = 13.01; *SD =* 1.15). From the all‐boys school, 143 students at T2 were excluded from data collection, due to disturbances and laxed discipline in some classrooms. From Grade 7 of one school, 185 students had received two sets of questionnaires during data collection at T1, one in English and the second in Hindi the next day, because the students found the English questionnaires difficult to follow on day one despite the medium of instruction for that school being English, thus excluding these students from final analyses. All students (337) of another of the 10 participating schools were excluded from the analyses as the school chose to drop out in Wave 3 because of undisclosed reasons, and, hence, data were missing, not at random. Five students were excluded due to incomplete data on their grades. Consequently, the final sample consisted of 1238 students from nine schools.

Beyond the abovementioned exclusions, students that opted out of the research or were absent during data collection (118 at T1; 202 at T2; and 232 at T3) were marked as missing in analyses. A distinction between who opted out and who was absent during data collection was not made in the present study. Descriptive statistics for age, socioeconomic status (SES), BMI, and victimization scores of the participants are reported in Table 1.

**Table 1:** Descriptive statistics for the main variables in the study

Variable	*N*	*M*	*SD*	Range
Age (T1)	1125	13.15	1.11	10
Age (T2)	1028	13.32	1.21	8
Age (T3)	1014	13.60	1.18	7
SES (T1)	1118	4.91	2.29	9
SES (T2)	1027	5.11	2.29	9
SES (T3)	995	5.17	2.25	9
BMI (T1)	1025	18.36	3.73	27.59
BMI (T2)	1023	18.53	3.71	29.89
BMI (T3)	954	18.57	3.83	28.22
Self‐report victim (T1)	1084	2.13	1.10	4.00
Self‐report victim (T2)	1014	2.16	1.13	4.00
Self‐report victim (T3)	987	2.18	1.13	4.00
Peer‐report victim (T1)	1233	16.49	19.97	94.44
Peer‐report victim (T2)	1235	28.89	19.11	80.00
Peer‐report victim (T3)	1236	26.72	15.93	88.89

Abbreviations: BMI, body mass index; SES, socioeconomic status.

### 2.2 Instruments

Students provided information regarding sociodemographics like gender, grade, age, and family affluence. The original English scales used in the present study were translated to Hindi, India's national language, through a formalized translation procedure following guidelines laid by Beaton et al. (2000). Three independent bilingual persons (high school teachers) living in Indore, forward translated the English scales to Hindi. The three persons then reviewed the discrepancies in the translated versions and synthesized a fourth unanimous version of the Hindi questionnaires. The Hindi version was then back‐translated to English by three other independent bilingual individuals who were not presented with the original English versions of the instruments beforehand. The two versions, forwarded translated Hindi as well as back‐translated English questionnaires, were compared with the original English scale to examine discrepancies in semantics as well as conceptualization. A pilot study was carried out with 60 students (not part of the main dataset) in English, and 60 in Hindi, before the start of the longitudinal data collection. Words identified as difficult to understand or unclear by students in both versions were carefully reviewed and edits were made by consensus of the bilingual translators and the first and third authors of the present study. A written report documenting the synthesis and issues addressed in the adaptation of the scales was maintained. Both, Hindi and English, scales were found to show good test–retest reliability as indicated in the paragraphs below.

#### 2.2.1 Family Affluence Scale II

The Family Affluence Scale II (FAS; Currie et al., 1997) was used to measure SES. This self‐report measure consists of four questions, each using a different response scale. FAS was developed so that adolescents can give an approximation of their SES. The FAS has been found to be a valid indicator of SES (Boyce et al., 2006), and has been validated for its use with Indian adolescents (Bapat, 2016). Test–retest correlations between Wave 1 and 2, Wave 2 and 3, and Wave 1 and 3 were found to be *r* = .73, *r* = .79, and *r* = .75 for the English questionnaires, and *r* = .70, *r* = .77, and *r* = .65 for the Hindi questionnaires.

#### 2.2.2 Self‐reported bullying victimization

The Illinois Bully‐Fight‐Victim Scale (Espelage & Holt, 2001) was used to assess self‐reported bullying and victimization. The scale has been found valid and reliable in western (Espelage et al., 2003), as well as Indian contexts (Sharma et al., 2020; Thakkar et al., 2020). We used data from the victim subscale for analyses. The victimization scale consists of four items that measure the experience of victimization from peers (e.g., “Other students picked on me”). Response options for the scales are (1) *never*, (2) *one or two times*, (3) *three or four times*, (4) *five or six time*s, and (5) *seven or more times* in the past 30 days. In the present study, Cronbach's *α* for this scale was found to be .81 at T1, .84 at T2, and .85 at T3 for the English questionnaires and .88 at T1, .90 at T2, and .92 at T3 for the Hindi questionnaires.

#### 2.2.3 Peer‐reported bullying victimization

All students were given a sheet of paper that described bullying behavior on the top in a few words (teasing, fighting, excluding, name‐calling, etc.), and had two columns with a list containing the first and last names of all classmates. Students were asked to nominate bullies (circle names in the first column) from their class and draw a line from the bullies to their victims in the second column. While the number of victims to be listed was not limited, we set a limit of up to five nominations for bullies to be listed. This was essential to avoid having the chaos of crossing lines and consequently scoring problems. Dyadic nominations of bully and victim status, received by peers from within a class, are found to be a reliable and valid estimate yielding consistent results with other informant reports across studies (Malamut et al., 2020; Veenstra et al., 2007) as well as in the Indian setting (Thakkar et al., 2020). A total score was computed based on the number of times an individual was marked as a victim by their classmates. This total score was changed into proportions by dividing the total score by the number of students in the class (Veenstra et al., 2007).

#### 2.2.4 Body mass index

Students' height (in centimeters) and weight (in kilograms) were measured objectively using standard weight and height equipment for each wave. Height in centimeters was converted into meters, and BMI was calculated using the formula BMI = weight (kg)/height^2^ (m) (Cole et al., 2000).

### 2.3 Procedure

The Institutional Review Board of the Institute of Education and Child Studies at Leiden University approved of the study. A convenience sample was obtained by approaching 15 schools in the school year 2015–2016. Ten schools agreed to participate. Monetary compensation was not offered to any participating school at the outset; however, of the four schools where the Principals requested it, either overtly, or during the conversation with the researchers, three schools were given compensation vouchers of a bookstore for each wave, whereas one school was given carpets for the students to sit on in the classroom. Participating students were not offered independent compensation, and students were told that their participation was voluntary and that their answers would not be shared with parents, teachers, or classmates. Furthermore, instructions to the students included that their participation in the research would bear no consequence on their academic performance, or have any other implications, neither positive nor negative. Regulations of research in India have been identified as different from the western context which does not necessarily reflect the requirements of India, especially given complex factors such as culture, level of parental education, demographics, and SES of participating schools in India (Bapat, 2016; Nijhawan et al., 2013). At the discretion and recommendation of the Principals of the participating schools, the principals, acting in loco parentis, gave written consent to collect data from students in participating Grades 7–9. Principals were informed of all the features of the research that may affect their willingness to allow the child to participate, and have been accepted in adolescent research to substitute in place of parents in school settings as responsible adults for children (Malamut et al., 2020). Parents were not invited to give consent, but students were allowed to opt out. Every student enrolled in a class at the participating schools was invited to complete the questionnaire. Most students present at the days of data collection chose to participate; however, some students chose to go to the library or complete their home assignments in the back rows of the class, thus resultingly being marked as absent (missing) in analyses. Thus, a record telling the absentees apart from the students who opted out was not maintained. All attention focused on the students filling out the questionnaires by addressing their questions and keeping them at task during data collection. Given that there was minimal risk in participation for the students in the present study, protection of confidentiality of the information provided, and the voluntary nature of participation where students could opt out of research, the present study deemed cogent support as per global ethical standards (Coyne, 2010; Tigges, 2003), and as seen in western research on bullying (O'Brien & Dadswell, 2020; Pickles, 2020), to conduct research with students by obtaining informed consent from participating students and Principals acting in loco parentis.

The questionnaires were distributed to the students in their classrooms during a prearranged time. There was a team of 20 trained research assistants, who were all first‐ or second‐year master's students of social work. During simultaneous data collection in multiple grades, at least two research assistants were present in each class, gave instructions, and were available to answer any of the students' questions. Students sat next to each other on benches and were instructed not to look at each other's responses and cover their questionnaires while filling them out. Class teachers helped to keep students on task but were asked not to interfere with completing the questionnaires. The students took approximately 75 min to complete the full questionnaire. Research assistants measured students' height and weight by asking each student to step outside the classroom, without removing their shoes. This step ensured that privacy was maintained while collecting information on student's height and weight, thereby protecting students' BMI information and allowing discretion. The data for height and weight measurements were kept confidential from other students and teachers.

### 2.4 Data analysis

For the self‐reported victim scale, at Step 1, we computed means for students who had responded to 80% or more items on the self‐reported bully/fight and victim subscales for T1, T2, and T3, respectively, while scale scores for students who had incomplete data on more than 20% items on each subscale in a particular wave were defined as missing. The 80% cutoff rule was in line with the criterion proposed by the authors of the scale (Espelage & Holt, 2001) and necessary as a first step to calculate a mean score for bullying victimization. This score was then used as a variable in the main analyses to examine a transactional model of influence between victimization and BMI. The missings as deduced through step 1 were handled using a full information maximum likelihood (FIML) estimation in the main analysis as explained in the next paragraph. For the peer‐reported victim scales, the percentage of times a child was marked a victim in class was calculated by classroom size (count × 100/total number of students in class) (Veenstra et al., 2007).

The transactional model was tested by conducting structural equation modeling analyses using *R version 3.5.1* (R Core Team, 2019). First, to test the unidirectional effects model in main analyses, concurrent associations, that is, explicit T1 to T2 to T3 factor loadings, between BMI and victimization were examined at baseline. We model these loadings explicitly to find the resulting residual covariance structure for model fit evaluation. Stability effects were investigated by studying regression lines between the same constructs over time. At Step 2, cross‐paths were added either from BMI to victimization or from victimization to BMI to test for longitudinal one‐way effects of BMI on victimization, or vice versa. Finally, to test the reciprocal‐effect model, both BMI to victimization and victimization to BMI cross‐paths were added to test bidirectional associations between BMI and victimization. Age and SES were accounted for as covariates in the transactional model. Separate analyses were conducted for gender, to examine differences in BMI and victimization association between boys and girls. Data were corrected for between‐subjects and within‐subjects dependence given the nested structure of the study. Correction of multicollinearity between variables in nested data is a procedure that, although needed, is underused in analyses in the studies of victimization (Bayaga, 2010). In the present study, both models for self‐ and peer‐reports, for each gender, had standard errors corrected for multilevel robustness to account for residual nesting effects, even though the intraclass correlation coefficients for both BMI and victimization ranged between 0.0154 and 0.0876, and can be considered negligible.

#### 2.4.1 Missing value analyses

Missing value analyses indicated that Little's (1988) missing completely at random (MCAR) was significant (*χ* ^2^ (424) = 670.14, *p* < .001). Data can only be tested for the assumption of MCAR. However, FIML estimation is a sophisticated procedure known to also adequately deal with data that are not MCAR, and, thus, all statistics reported in the analyses used the FIML estimation (Schlomer et al., 2010). In the transactional model, we used FIML because we computed subscale means for only those students who had responded to at least 80% items or more items on the self‐reported victimization scale, and data were still missing for students who had not responded on 80% of the items in certain waves. These missing values were dealt with using FIML estimations in main analyses which allow us to not only include students for whom we had mean scores at T1, T2, and T3, but also those students for whom we had means at both T1 and T2, but not T3, or students for whom we had means for both T2 and T3, but not T1, and, thus, students with less than 80% responses were also included in final analyses.

## 3 RESULTS

Descriptive statistics for the main variables in the study are reported in Table 1. Zero‐order correlations (Table 2) show significant positive correlations between BMI scores across time which confirms the stability of BMI. Furthermore, zero‐order correlations show that self‐reported victimization scores were positively interrelated across time points, and the same was true for peer‐reports. Concurrent associations between self‐ and peer‐reports victimization scores were weak, although significant, and interrater reliability between the self‐ and peer‐reports of victimization was not significant (Krippendorff's *α* > .05 at T1, T2, and T3) which indicates that self‐reported scores yield a different set of victims as compared to peer‐reported victims.

**Table 2:** Zero‐order correlations for variables in the study

	1	2	3	4	5	6	7	8	9
1. BMI (T1)	1								
2. BMI (T2)	.94^a^	1							
3. BMI (T3)	.94^a^	.96^a^	1						
4. Self‐report victim (T1)	.01	.02	0.6	1					
5. Self‐report victim (T2)	.04	.05	.02	.52^a^	1				
6. Self‐report victim (T3)	.06	.07	.05	.42^a^	.49^a^	1			
7. Peer‐report victim (T1)	.02	.05	.05	.12^a^	.10^a^	.09^a^	1		
8. Peer‐report victim (T2)	.04	.06	.06	.22^a^	.19^a^	.12^a^	.48^a^	1	
9. Peer‐report victim (T3)	−.08^a^	−.03	−.06^b^	.13^a^	.14^a^	.10^a^	.42^a^	.38^a^	1

Abbreviation: BMI, body mass index.

^a^ Correlation is significant at the .01 level (two‐tailed).

^b^ Correlation is significant at the .05 level (two‐tailed).

**Figure 1:**
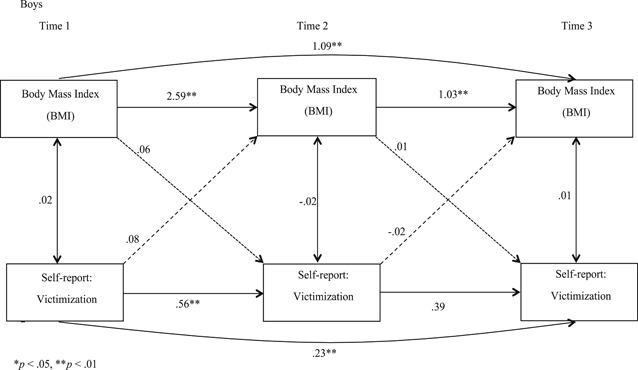
Transactional model of body mass index and self‐reported victimization for boys

**Figure 2:**
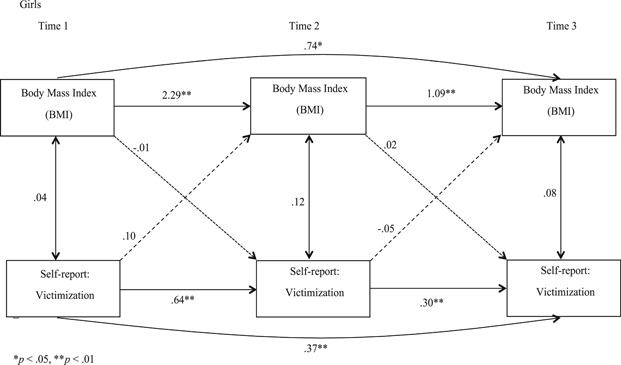
Transactional model of body mass index and self‐reported victimization for girls

**Figure 3:**
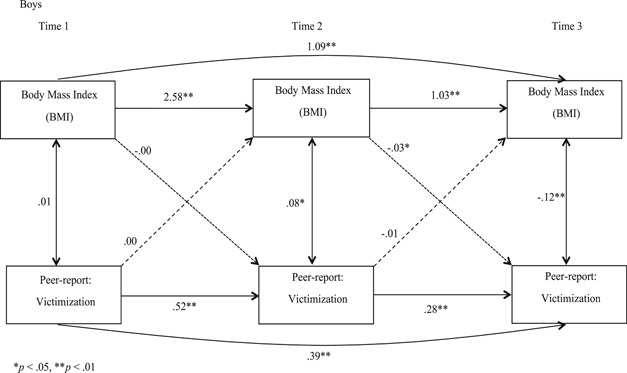
Transactional model of body mass index and peer‐reported victimization for boys

**Figure 4:**
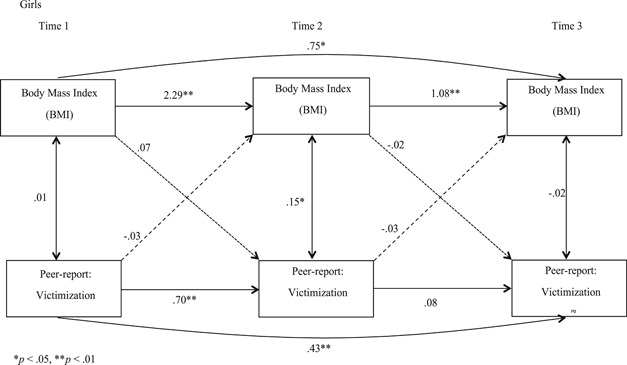
Transactional model of body mass index and peer‐reported victimization for girls

With SES and age added as covariates, we conducted separate analyses for boys and girls for self‐reported victimization (Figures 1 and 2) and peer‐reported victimization (Figures 3 and 4) to examine if BMI predicted victimization, and victimization predicted BMI concurrently, and over time. Stability effects show that BMI predicted BMI over time and victimization predicted victimization over time, for both the genders for self‐ as well as peer‐reports. However, for girls, we observed that peer‐reported victimization at T2 did not predict peer‐reported victimization at T3 (Figure 4), which we estimate to be a chance nonobservation given that stability effects are observed at other time points. When paths were added from BMI to victimization (dotted line), and victimization to BMI (dashed line), unidirectional effect findings indicated that for self‐reports, BMI did not predict victimization concurrently or over time for boys or girls, and vice versa. For peer‐reports, BMI and victimization were concurrently associated at T2 (*B =* .08, *p* < .05) and T3 (*B =* −.12, *p* < .01) for boys, and at T2 for girls (*B =* .15, *p* < .05). Also, BMI at T2 predicted victimization at T3 for boys (*B =* −.03, *p* < .05; Figure 3), indicating unidirectional effects over time running from BMI to victimization for peer‐reports. Furthermore, when cross‐paths were added to examine the reciprocal effect model from victimization at T1 to BMI at T2, and BMI at T2 to victimization at T3 with a direct path from victimization at T1 to victimization at T3, we observed that the bidirectional influence model was rejected for both genders for self‐ as well as peer‐reported victimization. Correlation coefficients reported in Figures 1, 2, 3, 4 are standardized and are all lagged coefficients.

## 4 DISCUSSION

Hypotheses of the present study were that there are concurrent and over time unidirectional effects, two‐term bidirectional associations between BMI and victimization, and three‐term reciprocal associations such that BMI predicts victimization which, in turn, predicts BMI, and vice versa. The hypotheses were rejected for boys and girls for self‐reported victimization. For peer‐reports, we observed concurrent, and unidirectional over time associations between BMI and victimization for boys, such that BMI at T2 predicts victimization at T3; however, the magnitude of the association is modest, and the direction negative. The reciprocal effect model is rejected for peer‐reports for both genders.

In the present study, we found no significant associations between BMI and self‐reported victimization for either gender, whereas peer‐reported victimization presented a different picture. Peer‐estimation procedures have been reported to be better identifiers of victims as compared to self‐estimation procedures in the study of bullying behaviors (Malamut et al., 2020; Salmivalli et al., 1996), but few studies used peer‐reports (Van Geel et al., 2017). With peer‐reports of victimization in the present study, we found two concurrent and one serial significant association between BMI and victimization, which is consistent with past literature (Baldwin et al., 2016; Janssen et al., 2004; Lee et al., 2018; Pearce et al., 2002). The association between BMI and victimization for boys at T2 is positive, whereas at T3 the same association is negative. Furthermore, the longitudinal unidirectional link where BMI at T2 predicts victimization at T3 for boys, also shows a negative direction of influence, that is, higher BMI leads to a decrease in victimization for boys over time. Similar findings have been reported in past research (Griffiths et al., 2006; Lee & Vaillancourt, 2019), where compared to average weight boys, some boys who are obese were more likely to be overt bullies, while other boys who are obese were more likely to be overt victims 1 year later, suggesting that BMI status has a mixed relationship with victimization among boys. Adolescent boys are more likely to engage in physical bullying as compared to girls (Smith & Ananiadou, 2003; Thakkar et al., 2020), and, hence, there may be some distinct advantage to being overweight or obese during adolescence for boys, as it may manifest physical dominance through greater strength, and the resulting popularity in the peer group may decrease their risk of victimization. If, nevertheless, they become victims, this could be because the boys deviate from appearance ideals or because they experience and show a lack of confidence in interactions with peers (Salmivalli & Peets, 2009). In the present study, we estimate that the change in the magnitude of concurrent associations between BMI and victimization, from positive at T2 to negative at T3 could also be due to the fact that victimization of students who are obese does not last long and the “joke just gets old,” or also, as observed in India, boys tend to learn to “deal with victimization on their own” (Erum, 2018).

Furthermore, for the reverse direction of influence, we found that there is no significant association running from victimization to weight gain, for either of the genders, for self‐ and peer‐reported victimization. Similar conclusions have been observed in past longitudinal studies (Lumeng et al., 2010), where nonsignificant relation between victimization and BMI could mean that peer victimization does not play a direct role in influencing BMI, but it is influenced by other, mediating factors, or a combination of several of them, like self‐esteem or stress‐eating habits (Giletta et al., 2010; Lee et al., 2017), or through body dissatisfaction as found in recent research (Lee & Vaillancourt, 2018, 2019; Lin et al., 2018; Lunde et al., 2007). It has been found that the relationship between overweight and experiencing physical and relational bullying seems to be mediated by factors associated with a child's weight status like global self‐worth, self‐esteem for physical appearance, and body dissatisfaction (Fox & Farrow, 2009). Brixval et al. (2012) notably confirmed in their study that the relationship between adolescents' weight status and bullying is completely mediated by the role of body image, similar to which, the longitudinal study by Lee and Vaillancourt (2019) found that peer victimization had direct and indirect effects on BMI longitudinally, via body dissatisfaction.

We hypothesized cross‐cultural associations between BMI and victimization based on results from previous studies (Van Geel et al., 2014). However, we do not find similar results with self‐reports in the present study, and even with peer‐reports, the findings are not consistent for each observation during the three time points. One possible explanation for the findings of the present study could be that appearance standards among Indian adolescents are not the same as in western countries. For example, in contemporary Indian society, a protruding belly speaks of a life of “*embodied satisfaction – good social relationships, status, success and health*” (Wilson, 2010). Pells et al. (2016) indicate that in non‐western countries like India, not all contributory factors associated with bullying are necessarily linked with structural factors of their associations. For example, in India, “thinness” may reflect malnutrition due to poverty, or obesity may reflect the social elitism of well‐fed affluent families. Furthermore, binge eating is not a habit commonly observed among Indian adolescents, because “indulgence” is seen as a *moral* misdeed among the nonupper class people of India and is often criticized in contemporary society (Wilson, 2010). Thus, the present study emphasizes the need to further examine the variables of BMI and victimization and the association between the two within the *context* of the Indian culture.

### 4.1 Limitations and conclusion

The present study has limitations. We do not differentiate between the different forms of victimization experiences (physical, social, or relational) (Janssen et al., 2004) in the present study, and, hence, we cannot speak of their specific associations with BMI. A second limitation of the present study is the considerable number of exclusions made to data due to logistical and administrative challenges encountered during data collection that, although commonly observed in India (Bapat, 2016; Thakkar et al., 2020), may have contributed to the possibility that there were important factors that got missed out in the present study. To this end, the present study maintains transparency in the reporting of exclusions and strengthens methodological rigor in analyses to overcome this limitation. We conclude that BMI shows a small prospective effect and some concurrent effects in different directions on victimization for boys; however, overall the transactional model of BMI and victimization is not supported in India.

### 4.2 Implications and directions for future research

Despite the limitations of the present study, the findings are important for school health. This study, while using a rigorous longitudinal design with both self‐ and peer‐reports of bully victimization as well as objective measures of BMI shows that results from previous western prospective studies about BMI and victimization cannot be generalized to an urbanized area in India. Because the results from western studies may not be generalizable, professionals in India, with 356 million youth the largest youth population in the world (UNFPA, 2014), cannot build their preventions and interventions on the knowledge base about precursors and consequences of bullying available in the western world. This calls for new research. Part of the research should again focus on cross‐validation especially considering that research findings from western studies are used, rather presumptuously, to design interventions in lower‐income countries where indigenous research is sparse (Kalra et al., 2012; Thakkar et al., 2020). School interventions and policies need to go beyond the assumption of peer victimization as a risk factor in predicting weight gain over time, by examining context‐specific variables and cultural factors, appearance ideals, and eating habits in India as compared to western countries, in addition to victimization experiences at school (Pells et al., 2016).

## CONFLICT OF INTERESTS

The authors declare that there are no conflict of interests.

## Data Availability

The data that support the findings of this study are available from the corresponding author upon reasonable request.
